# Supplementary material for: Unveiling the Bioactive Potential of the Invasive Jellyfish Phyllorhiza punctata Through Integrative Transcriptomic and Proteomic Analyses
Source: Biomolecules. 2025 Aug 4;15(8):1121. doi: 10.3390/biom15081121 (PMC12383608; doi:10.3390/biom15081121)
Supplement: Supplementary file 1 [file biomolecules-15-01121-s001.zip › Suplementary Material/FileS3/PEPFOLD4/XP_065058797_15282/clusters.html]

 PEP-FOLD clusters


Download archive of all models  here   

|  |  |  |
| --- | --- | --- |
| # | file | sOPEP |
| # cluster 1 - Size: 61 |  |  |
|  | XP\_065058797\_15282-model1 | -12.3712 |
|  | XP\_065058797\_15282-model1.1 | -12.3712 |
|  | XP\_065058797\_15282-model1.2 | -12.3712 |
|  | XP\_065058797\_15282-model1.3 | -12.3712 |
|  | XP\_065058797\_15282-model1.4 | -12.3712 |
|  | XP\_065058797\_15282-model1.5 | -12.3712 |
|  | XP\_065058797\_15282-model1.6 | -12.3712 |
|  | XP\_065058797\_15282-model1.7 | -12.3712 |
|  | XP\_065058797\_15282-model1.8 | -12.3712 |
|  | XP\_065058797\_15282-model1.9 | -12.3712 |
|  | XP\_065058797\_15282-model1.10 | -12.3712 |
|  | XP\_065058797\_15282-model1.11 | -12.3712 |
|  | XP\_065058797\_15282-model1.12 | -12.3712 |
|  | XP\_065058797\_15282-model1.13 | -12.3712 |
|  | XP\_065058797\_15282-model1.14 | -12.3712 |
|  | XP\_065058797\_15282-model1.15 | -12.3712 |
|  | XP\_065058797\_15282-model1.16 | -12.3712 |
|  | XP\_065058797\_15282-model1.17 | -12.3712 |
|  | XP\_065058797\_15282-model1.18 | -12.3712 |
|  | XP\_065058797\_15282-model1.19 | -12.3712 |
|  | XP\_065058797\_15282-model1.20 | -12.3712 |
|  | XP\_065058797\_15282-model1.21 | -12.3712 |
|  | XP\_065058797\_15282-model1.22 | -12.3712 |
|  | XP\_065058797\_15282-model1.23 | -12.3712 |
|  | XP\_065058797\_15282-model1.24 | -12.3712 |
|  | XP\_065058797\_15282-model1.25 | -12.3712 |
|  | XP\_065058797\_15282-model1.26 | -12.3712 |
|  | XP\_065058797\_15282-model1.27 | -12.3712 |
|  | XP\_065058797\_15282-model1.28 | -12.3712 |
|  | XP\_065058797\_15282-model1.29 | -12.3712 |
|  | XP\_065058797\_15282-model1.30 | -12.3712 |
|  | XP\_065058797\_15282-model1.31 | -12.3712 |
|  | XP\_065058797\_15282-model1.32 | -12.3712 |
|  | XP\_065058797\_15282-model1.33 | -12.3712 |
|  | XP\_065058797\_15282-model1.34 | -12.3712 |
|  | XP\_065058797\_15282-model1.35 | -12.3712 |
|  | XP\_065058797\_15282-model1.36 | -12.3712 |
|  | XP\_065058797\_15282-model1.37 | -12.3712 |
|  | XP\_065058797\_15282-model1.38 | -12.3712 |
|  | XP\_065058797\_15282-model1.39 | -12.3712 |
|  | XP\_065058797\_15282-model1.40 | -12.3712 |
|  | XP\_065058797\_15282-model1.41 | -12.3712 |
|  | XP\_065058797\_15282-model1.42 | -12.3712 |
|  | XP\_065058797\_15282-model1.43 | -12.3712 |
|  | XP\_065058797\_15282-model1.44 | -12.3712 |
|  | XP\_065058797\_15282-model1.45 | -12.3712 |
|  | XP\_065058797\_15282-model1.46 | -12.3712 |
|  | XP\_065058797\_15282-model1.47 | -12.3712 |
|  | XP\_065058797\_15282-model1.48 | -12.3712 |
|  | XP\_065058797\_15282-model1.49 | -12.3712 |
|  | XP\_065058797\_15282-model1.50 | -12.3712 |
|  | XP\_065058797\_15282-model1.51 | -12.3712 |
|  | XP\_065058797\_15282-model1.52 | -12.3712 |
|  | XP\_065058797\_15282-model1.53 | -12.3712 |
|  | XP\_065058797\_15282-model1.54 | -12.3712 |
|  | XP\_065058797\_15282-model1.55 | -12.3712 |
|  | XP\_065058797\_15282-model1.56 | -12.3712 |
|  | XP\_065058797\_15282-model1.57 | -12.3712 |
|  | XP\_065058797\_15282-model1.58 | -12.3712 |
|  | XP\_065058797\_15282-model1.59 | -12.3712 |
|  | XP\_065058797\_15282-model1.60 | -12.3712 |
| # cluster 2 - Size: 5 |  |  |
|  | XP\_065058797\_15282-model2 | -8.13031 |
|  | XP\_065058797\_15282-model2.1 | -8.13031 |
|  | XP\_065058797\_15282-model2.2 | -8.13031 |
|  | XP\_065058797\_15282-model2.3 | -8.13031 |
|  | XP\_065058797\_15282-model2.4 | -8.13031 |
| # cluster 3 - Size: 27 |  |  |
|  | XP\_065058797\_15282-model3 | -7.1857 |
|  | XP\_065058797\_15282-model3.1 | -7.06276 |
|  | XP\_065058797\_15282-model3.2 | -7.06276 |
|  | XP\_065058797\_15282-model3.3 | -7.06276 |
|  | XP\_065058797\_15282-model3.4 | -7.06276 |
|  | XP\_065058797\_15282-model3.5 | -7.03275 |
|  | XP\_065058797\_15282-model3.6 | -7.03275 |
|  | XP\_065058797\_15282-model3.7 | -7.03275 |
|  | XP\_065058797\_15282-model3.8 | -7.03275 |
|  | XP\_065058797\_15282-model3.9 | -7.03275 |
|  | XP\_065058797\_15282-model3.10 | -7.00755 |
|  | XP\_065058797\_15282-model3.11 | -7.00755 |
|  | XP\_065058797\_15282-model3.12 | -7.00755 |
|  | XP\_065058797\_15282-model3.13 | -7.00755 |
|  | XP\_065058797\_15282-model3.14 | -6.87411 |
|  | XP\_065058797\_15282-model3.15 | -6.74678 |
|  | XP\_065058797\_15282-model3.16 | -6.74678 |
|  | XP\_065058797\_15282-model3.17 | -6.63546 |
|  | XP\_065058797\_15282-model3.18 | -6.63295 |
|  | XP\_065058797\_15282-model3.19 | -6.49964 |
|  | XP\_065058797\_15282-model3.20 | -6.4241 |
|  | XP\_065058797\_15282-model3.21 | -6.37486 |
|  | XP\_065058797\_15282-model3.22 | -6.21153 |
|  | XP\_065058797\_15282-model3.23 | -5.98946 |
|  | XP\_065058797\_15282-model3.24 | -5.899 |
|  | XP\_065058797\_15282-model3.25 | -5.899 |
|  | XP\_065058797\_15282-model3.26 | -5.54157 |
| # cluster 4 - Size: 4 |  |  |
|  | XP\_065058797\_15282-model4 | -6.8329 |
|  | XP\_065058797\_15282-model4.1 | -5.93725 |
|  | XP\_065058797\_15282-model4.2 | -5.44146 |
|  | XP\_065058797\_15282-model4.3 | -5.24582 |
| # cluster 5 - Size: 2 |  |  |
|  | XP\_065058797\_15282-model5 | -6.25065 |
|  | XP\_065058797\_15282-model5.1 | -6.11971 |
| # cluster 6 - Size: 1 |  |  |
|  | XP\_065058797\_15282-model6 | -5.52767 |
|  |  |  |
